# Low-Grade Appendiceal Mucinous Neoplasm: What Is the Best Treatment?

**DOI:** 10.7759/cureus.46591

**Published:** 2023-10-06

**Authors:** Murat Guner, Cengiz Aydın

**Affiliations:** 1 Department of General Surgery, University of Health Sciences Izmir Tepecik Education and Research Hospital, Izmir, TUR

**Keywords:** oncologic surgeries, mucinous neoplasms, pseudomyxoma peritonei, appendix, low-grade mucinous neoplasm, lamn

## Abstract

Background: Low-grade appendiceal mucinous neoplasm (LAMN) is an uncommon tumor of the appendix that is usually diagnosed incidentally after surgery. Although LAMN may be asymptomatic, it can rupture and seed mucin and neoplastic epithelium into the peritoneum, causing pseudomyxoma peritonei (PMP).

Materials and methods: Data from 53 patients were retrospectively analyzed. Age, sex, tumor size, margin status, peritoneal carcinomatosis index, surgical procedures, postoperative results with histologic diagnosis, T stage, recurrence, and mortality of the patients were evaluated.

Results: Appendectomy was performed in 37 patients, right hemicolectomy in nine patients, cytoreductive surgery in one patient, and cytoreductive surgery and hyperthermic intraperitoneal chemotherapy in six patients. Recurrence occurred in four patients. Of the patients who developed recurrence, one patient had stage T4a disease, and the other three patients had T4b disease (p<0.001). Eighteen patients had acellular mucin in the serosa and four of these patients developed recurrence (p=0.004). Twelve patients had appendix perforation, and three of these patients had PMP on exploration (p<0.001). The mean survival time was 93.3 months in patients without recurrence and 32 months in patients with recurrence (p=0.021).

Conclusions: Low-grade appendiceal mucinous neoplasms are rare appendiceal tumors. The appropriate management of this tumor is controversial. T stage, appendix perforation, presence of acellular mucin on the serosa, and surgical margins are risk factors for the development of PMP. Appendectomy is considered sufficient when there are no risk factors for Tis (LAMN) and T3 disease. Right hemicolectomy may be sufficient if there are no risk factors for T4a disease, but cytoreductive surgery and hyperthermic intraperitoneal chemotherapy seem to be the most appropriate treatments in the presence of the stated risk factors for T4b disease.

## Introduction

Mucinous neoplasia of the appendix is a rare condition that occurs in approximately 1% of the patients undergoing appendectomy [1]. Low-grade appendiceal mucinous neoplasms (LAMN) are characterized by low-grade cytologic features, invasion pattern of appendiceal layers, villous or flat proliferative intestinal-type mucinous epithelium, significant mucin production, and typical limitations of the muscularis propria [2]. Patients with LAMN often show appendicitis-like symptoms such as pain in the right iliac fossa, fever, nausea, and vomiting [3]. Depending on the increase in mucin in the appendix lumen, it may present as a palpable mass or may be diagnosed after appendectomies are performed due to appendiceal abnormalities in surgeries performed for other reasons [4]. In more advanced stages, patients can present with intermittent colicky pain, intestinal obstruction due to mass effects, and genitourinary symptoms due to obstruction of the ureter [5].

Patients with LAMN without perforation have a better prognosis with curative surgery, but the presence of perforation causes the intraperitoneal spread of neoplastic cells and mucinous acid, resulting in pseudomyxoma peritonei (PMP) [6]. Patients may not have any acute abdominal pain associated with tumor rupture; however, increased mucus accumulation due to tumor implantation causes abdominal swelling, discomfort, pain, and palpable abdominal masses.

There is little agreement on the treatment of a subset of patients with LAMN with intermediate clinicopathologic highlights, such as localized disease or the presence of acellular mucin on the serosa [7]. The treatment of LAMNs confined to the appendix remains controversial. Some researchers believe that preventive extended resection is not beneficial for the survival of patients with negative appendectomy margins [8]. In advanced patients, cytoreductive surgery (CRS) and hyperthermic intraperitoneal chemotherapy (HIPEC) are recommended; however, the benefit of prophylactic CRS+HIPEC in high-risk patients without peritoneal spread is controversial [9].

In this study, we aimed to investigate the surgical procedures performed in patients with LAMN, histopathological findings, and risk factors for recurrence.

## Materials and methods

A total of 6849 patient files were analyzed retrospectively between January 2012 and December 2022; 72 patients had LAMN. Fourteen patients with missing data and five patients who did not attend follow-up were excluded from the study. As a result, 53 patients diagnosed with LAMN were included in the study. Patient's age, sex, tumor size, margin status, peritoneal carcinomatosis index (PCI), surgical procedures, postoperative results with histological diagnosis, T stage, recurrence, and mortality were evaluated.

Only patients with pathological diagnoses of low-grade appendix mucinous neoplasia were included in the study. Thirty-two (60.3%) patients presented with emergencies, of which 28 patients underwent appendectomy for suspected acute appendicitis and four underwent right hemicolectomy for a mass in the cecum. Elective surgery was performed in 21 (39.6%) patients. LAMN was observed in eight patients who underwent appendectomy because of abnormal appendices in patients who underwent surgery for gynecologic pathologies. Elective appendicectomy was performed in four patients, and elective right hemicolectomy was performed for suspicious cecal masses in five patients. One CRS and three CRS plus HIPEC treatments were administered to patients who were diagnosed with PMP. Right hemicolectomy, total peritonectomy, omentectomy, and, if necessary, additional organ resections were performed in patients who underwent CRS+HIPEC. Oxaliplatin (360 mg/m2) was administered to the patients for 60 min or mitomycin C (40 mg/m2) for 120 min as an intraperitoneal chemotherapy. The first evaluation was performed 10 days after discharge, and patients were called for follow-up at the 1st, 3rd, and 6th months, respectively. In the 6th month, the patients were evaluated using abdominal computed tomography (CT) and followed up regularly.

Statistical analyses

Statistical analyses were performed using Statistical Package for the Social Sciences (SPSS) version 22.0 (IBM Corp., Armonk, NY). Categorical variables were presented as frequencies and percentages. Comparisons between the groups in terms of distribution were performed using Pearson’s Chi-square and Fisher’s exact tests. Univariate comparisons of statistics and clinical characteristics were performed using the Student's t-test. Kolmogorov-Smirnov and Levene tests were performed to assess the homogeneity and normality of the data. Thus, throughout the text, the continuous variables are presented as medians (minimum-maximum). Continuous variables between the two groups were compared using the Mann-Whitney U test. Kaplan-Meier survival analysis was performed to determine overall survival (OS). A p-value less than or equal to 0.05 was considered statistically significant.

## Results

Data from 53 patients were analyzed, of which 29 (55.7%) were male and 24 (45.3%) were female. The mean±standard deviation (SD) age of the patients was 60.30±14.02 (range, 22-88) years. The demographic distribution of the patients is shown in Table 1.

**Table 1:** Demographic distribution of all patients SD, standard deviation; Min, minimum; Max, maximum; CRS, cytoreductive surgery; HIPEC, hyperthermic intraperitoneal chemotherapy; PCI, peritoneal cancer index; DFS, disease-free survival

Age, year, mean±SD, range	60.30±14.02 (22-88)
Sex: n (%)	
Male	29 (55.7%)
Female	24 (45.3%)
Focal High-Grade Dysplasia: n (%)	
Absent	48 (90.6%)
Present	5 (9.4%)
Surgical Margin: n (%)	
Negative	49 (92.5%)
Positive	4 (7.5%)
Surgical margin distance, cm, mean±SD, range	4.17±5.01 (0-18)
Acellular Mucin: n (%)	
Absent	35 (66%)
Present	18 (34%)
Perforation: n (%)	
Absent	41 (77.4%)
Present	12 (22.6%)
T Stage: n (%)	
Tis	16 (30.2%)
T3	16 (30.2%)
T4a	14 (26.4%)
T4b	7 (13.2%)
Tumor size, cm, mean±SD, range	2.66±1.73 (1-9)
Peritoneal Carcinomatosis: n (%)	
Absent	46 (86.8%)
Present	7 (13.2%)
Surgical Procedure: n (%)	
Appendectomy	37 (69.8%)
Right hemicolectomy	9 (17%)
CRS	1 (1.9%)
CRS+HIPEC	6 (11.3%)
PCI, mean±SD, range	13.57±8.24 (3-26)
Recurrence: n (%)	
Absent	49 (92.5%)
Present	4 (7.5%)
DFS time, month, mean±SD, range	39.7±23.56 (8-108)
Survey: n (%)	
Dead	45 (84.9%)
Alive	8 (15.1%)
Follow-up time, month, mean±SD, range	40.15±23.29 (8-108)

Appendectomy was performed in 37 patients, right hemicolectomy in nine patients, CRS in one patient, and CRS+HIPEC in six patients. Five patients had focal high-grade dysplasias. Surgical margins were positive in four (7.5%) patients. Three of these patients had PMP during exploration, and appendectomy was performed diagnostically. The other patient had a pathologic appearance only in the appendix, and the final pathology of this patient was evaluated as T4a. For the patient who had a positive surgical margin but no peritoneal disease was detected on exploration, only right hemicolectomy was performed, and no recurrence developed in the follow-up. Acellular mucin was found in the appendiceal serosa in 18 patients (34%). Appendicitis perforation was observed in 12 (22.6%) patients. According to the T stage, 16 (30.1%) patients had Tis, 16 (30.1%) had T3, 14 (26.4%) had T4a, and seven (13.2%) had T4b. The mean tumor size was 2.66±1.73 (range, 1-9) cm. PMP was present in seven (13.2%). The mean PCI was 13.57±8.24 (range, 3-26) in patients with PMP. The mean disease-free survival (DFS) was found as 39.7±23.56 (range, 8-108) months. Mortality was observed in eight patients during follow-up, but only two patients died of the disease.

Four patients had recurrence: three were female and one was male (p=0.214). The associations between recurrence and clinicopathological factors are shown in Table 2.

**Table 2:** Association between recurrence and clinicopathologic factors χ2: Pearson's Chi-square Test; t: Student’s t-test; U: Mann-Whitney U test, SD, standard deviation; Min, minimum; Max, maximum; CRS, cytoreductive surgery; HIPEC, hyperthermic intraperitoneal chemotherapy; PCI, peritoneal cancer index; DFS, disease-free survival

Clinicopathologic factors	No. of patients (%)		P value
	Recurrence (-)	Recurrence (+)	
	(49 patients, 92.5%)	(4 patients, 7.5%)	
Age, year, mean±SD, range	59.45±14.12 (22-88)	70.75±7.67 (62-79)	p=0.122 ^t^
Sex: n (%)			p=0.214^ x2^
Male	28	1	
Female	21	3	
Focal High-Grade Dysplasia: n (%)			p=0.502^ x2^
Absent	44	4	
Present	5	0	
Surgical Margin: n (%)			p=0.001^ x2^
Negative	47	2	
Positive	2	2	
Surgical margin distance, cm, median, range	2 (0-18)	0.5 (0-15)	p=0.117 ^u^
Acellular Mucin: n (%)			p=0.004^ x2^
Absent	35	0	
Present	14	4	
Perforation: n (%)			p<0.001^ x2^
Absent	41	0	
Present	8	4	
Seroza invasion (Stage T4): n (%)	(49 patients, 92.5%)	(4 patients, 7.5%)	p=0.010^ x2^
Absent	32	0	
Present	17	4	
Tumor size, cm, median, range	2 (1-9)	3.5 (2-7)	p=0.224^ u^
Peritoneal Carcinomatosis: n (%)			p<0.001^ x2^
Absent	45	1	
Present	4	3	
PCI, mean±SD	12.25±9.87 (3-26)	15.33±7.02 (8-22)	p=0.667 ^t^
Neoadjuvant Chemotherapy: n(%)			p<0.001^ x2^
Absent	48	1	
Present	1	3	
Surgical procedure: n (%)			p=0.002^ x2^
Appendectomy	36	1	
Right hemicolectomy	9	0	
CRS	0	1	
CRS+HIPEC	4	2	
DFS time, month, median, range	36 (8-108)	24 (18-32)	p=0.195^ u^
Follow-up time, month, median, range	36 (8-108)	31 (26-34)	p=0.429^ u^

Focal high-grade dysplasia was not observed in patients who developed recurrence. Two of the four patients with recurrence had positive surgical margins at the first diagnosis (p=0.001). In patients who underwent appendectomy, the median distance of the surgical margin was 2 cm (range, 0-18) cm in those without recurrence, and 0.5 (range, 0-15) cm in those with recurrence (p=0.117). Recurrence occurred in four of the 18 patients with acellular mucin in the serosa (p=0.004). Twelve patients had appendix perforation, three of whom had PMP on exploration (p<0.001). Recurrence was observed in four patients who developed perforation after appropriate surgical treatment. Of the patients who developed recurrence, one patient was in stage T4a and the other three patients had T4b (p<0.001). Recurrence was observed in one of the patients who underwent appendectomy and in none of the patients who underwent right hemicolectomy. In addition, recurrence occurred in one patient treated with CRS and two patients treated with CRS+HIPEC (p=0.002). The data of patients with recurrence are shown in Table 3.

**Table 3:** Data of patients with recurrence CRS, cytoreductive surgery; HIPEC, hyperthermic intraperitoneal chemotherapy; PCI, peritoneal cancer index

	Sex	Age	T stage	Perforation Yes/No	PCI	Surgical procedure	Exitus Yes/No
Patient 1	Female	67	T4b	Yes	22	CRS+HIPEC	Yes
Patient 2	Male	79	T4b	Yes	16	CRS	Yes
Patient 3	Female	75	T4b	Yes	8	CRS+HIPEC	No
Patient 4	Female	47	T4a	Yes	0	Appendectomy	No

The mean DFS was 39.7±23.56 (range, 8-108) months, and eight patients died during the follow-up period, but only two died of disease-related causes. The mean survival time was 93.3 months in patients without recurrence and 32 months in patients with recurrence (p=0.021).

## Discussion

In this study, which was conducted with 53 patients, LAMN confined to the appendix wall posed no risk for PMP, but a significant risk was found in patients with T4 disease.

There are studies stating that advanced age and female sex are risk factors for appendix tumors; however, in our study, no significant difference was found between the sexes [10-11]. The mean age was found as 60.30±14.02 (range, 22-88) years in patients with LAMN, but it was not statistically significant in patients with PMP, although the mean age was higher in those with PMP (70.75±7.67{62-79}).

There is a treatment algorithm for patients with LAMN in the literature, but it is not always possible to apply these algorithms because there may be an urgent need for surgery when patients present with acute abdomen symptoms [12]. The presence of mucoceles can be an important finding in preoperative examinations, but there are studies in the literature reporting that approximately 40% have been diagnosed preoperatively as acute appendicitis on clinical grounds alone [13]. In our study, 32 patients underwent emergency surgeries. Major surgery should be avoided in patients with no pathological diagnoses and suspected appendix mucinous neoplasia on exploration, and appendectomy would be more appropriate for diagnosis in these patients. In our study, no recurrence was observed in any patient who underwent right hemicolectomy, and only one patient who underwent appendectomy had PMP (p=.002). Gonzalez-Moreno and Sugarbaker described a method called radical appendectomy for incidental mucinous appendix neoplasms [14]. In the literature, right hemicolectomy performed without HIPEC preparation is not recommended because it may cause the disease to reach the retroperitoneum and increase the risk of recurrence in the anastomosis line [15-16]. In this study, 11 of 14 patients with T4a underwent appendectomy, three underwent right hemicolectomy, and recurrence was observed in only one patient who underwent appendectomy. An appendectomy may be sufficient in patients with limited LAMN in the appendix wall, but right hemicolectomy seems to prevent the development of PMP in limited disease.

Positive margins were found in four patients, and PMP was present in two of these patients (p=0.001). Among the patients with positive margins, right hemicolectomy was performed in two patients, CRS was performed in one patient, and CRS+HIPEC was performed in one patient with positive surgical margins. PMP developed in two of these patients who underwent CRS and CRS+HIPEC during follow-up, and these patients had T4b. In the study by Arnason et al., the surgical margin was positive in 15 patients; additional surgery was performed on six of the patients with positive surgical margins and not performed in nine patients, and no recurrence developed in either group [17]. Although surgical margin positivity was seen as a risk factor for PMP in our study, right hemicolectomy is feasible in patients with a disease limited to the appendix wall and no peritoneal disease. Surgical margin positivity is an important risk factor for recurrence in cancer surgery, and surgical margins are an important prognostic factor in appendiceal tumors. However, further studies are needed to determine the effect of surgical margins on the development of PMP in patients with LAMN.

Acellular mucin was found on the appendiceal serosa in 18 (34%) patients and four of these patients had PMP (p=0.004). Fournier et al. indicated the presence of acellular mucin on the serosa as a low-grade mucinous neoplasm of the appendix with uncertain malignant potential [18]. In our study, the presence of acellular mucin was found to be associated with PMP; however, only one of the 11 patients with T4a disease with acellular mucin in their serosa developed PMP.

Perforation of the appendix is one of the most important factors determining the treatment of mucinous neoplasia; however, Ballatine et al. reported that perforation was not associated with disease progression in T4a cases [19]. For non-perforated early stages of LAMN, appendectomy seems to be an adequate treatment; however, in advanced stages, appendectomy, right hemicolectomy, peritonectomy, and additional HIPEC should be considered (11). The Peritoneal Surface Oncology Group International (PSOGI) stated that CRS+HIPEC treatment could be considered in the presence of perforation in LAMN [12]. In our study, 12 patients had appendix perforation, which was associated with the development of PMP (p<0.001).

The mean maximal diameters of the masses ranged from approximately 1 cm to 9 cm, with a mean size of 2.66 cm in our series. However, we found no significant difference between the tumor size and PMP.

In our study, seven patients had PMP and the mean PCI was 13.57±8.24 (range, 3-26). Six of these patients underwent CRS+HIPEC, but one patient who underwent surgery for a gynecologic malignancy had only CRS, and LAMN was detected as a result of the definitive pathology of this patient. No statistically significant relationship was found between PCI and recurrence (p=0.667). PCI is an important parameter that determines prognosis in the treatment of peritoneal carcinomatosis; however, the complete cytoreduction score is more important [12]. The PSOGI does not consider a high PCI index an absolute contraindication for patients with LAMN [12]. In another study, Yuksel et al. reported that patients with a PCI of >17 could be managed by CRS/HIPEC if a CC score of ≤2 could be achieved [20]. In our study, recurrence was observed in two of the six patients who underwent CRS+HIPEC. Although the number of cases was insufficient to draw firm conclusions, it was observed that adding HIPEC to the treatment regimen contributed to patient prognosis.

The mean DFS was 39.7±23.56 (range, 8-108) months, and eight patients died during the follow-up period, but only two of these patients died of disease-related reasons. The mean survival time was 93.3 months in patients without recurrence and 32 months in those with recurrence (p=0.021). In one study, the 3-year DFS and OS rates were 72.6% and 91.6%, respectively, in patients with LAMN [21]. Solomon et al. reported that the 1-, 3-, and 5-year DFS rates in patients with LAMN were 95.5%, 83.4%, and 78.3%, respectively [22]. The high DFS rate is because the disease is treatable when detected at an early stage, and successful results are obtained with complete CRS and HIPEC treatment in cases of peritoneal spread. There are some limitations of this study. Due to the rarity of LAMN and the relatively low number of patients, more patients and studies are needed for subgroup analyses of patients with recurrence.

## Conclusions

LAMNs limited to the appendix lumen do not show definitive malignant features; however, when they exceed the appendix wall, they can show malignant features and cause PMP. In this study, T stage, appendix perforation, presence of acellular mucin on the serosa, and possitive surgical margins were risk factors for the development of PMP. In LAMN patients with T3 stage and below, appendectomy may be considered as an appropriate treatment option if there are no risk factors. Right hemicolectomy may be sufficient if there are no risk factors for T4a disease, but CRS and HIPEC seem to be the most appropriate treatments in the presence of the stated risk factors or T4b disease.
